# Sarcoidosis Presenting With Nephritis and Pancreatitis in a Pediatric Patient: A Rare Case

**DOI:** 10.7759/cureus.70635

**Published:** 2024-10-01

**Authors:** Njood Nazer, Reem A Al Zahrani, Najla Alotaibi, Emtenan Basahl, Mohammed Nashawi

**Affiliations:** 1 Pediatrics, King Fahad Military Medical Complex, Jeddah, SAU; 2 Medicine, King Abdulaziz University Faculty of Medicine, Jeddah, SAU; 3 Pediatric Nephrology Center of Excellence, King Abdulaziz University Hospital, Jeddah, SAU; 4 Medicine, King Abdulaziz University Hospital, Jeddah, SAU; 5 Pediatrics, King Abdulaziz University Faculty of Medicine, Jeddah, SAU

**Keywords:** ace, autoimmune, nephritis, pancreatitis, sarcoidosis

## Abstract

Sarcoidosis is a multisystem granulomatous disorder of undetermined etiology that usually affects the lungs. It is a rare presentation to have an association between sarcoidosis and pancreatitis. We present a challenging case of acute pancreatitis and kidney dysfunction which had been diagnosed later to be sarcoidosis. A 12-year-old boy with abdominal pain and weight loss for one month, however, had no respiratory manifestations. Investigations revealed elevated lipase and creatinine. The patient was managed for acute kidney injury with an initial suspicion for autoimmune pancreatitis. Further workup revealed acute interstitial nephritis with non-necrotizing granuloma on renal biopsy. He developed uveitis and was found to have a variant in the angiotensin-converting enzyme (ACE) gene, which led to the diagnosis of sarcoidosis. According to our knowledge, this is the first case with juvenile sarcoidosis involving the pancreas and kidney.

## Introduction

Sarcoidosis is a multisystem granulomatous disorder with an undetermined underlying cause that most often affects the lungs and may cause significant morbidity. This disease is characterized by T-lymphocyte infiltration, granuloma formation, and microarchitecture distortion [1]. The pathogenesis results from an uncontrolled cell-mediated immune reaction, manifested by well-formed, noncaseating epithelioid granulomas [2]. Most commonly, sarcoidosis affects individuals in their third to fourth decade of life, but it can affect anyone at any age [3]. Despite a large, well-matched, multicenter etiologic study, no single predominant environmental or occupational factor has been identified, although modest risk was found for exposure to moldy environments [4]. More than 30% of patients suffer from extrapulmonary sarcoid, which affects the skin, eyes, reticuloendothelial system, musculoskeletal system, exocrine glands, heart, kidney, and nervous system [5].

Patients with extrapulmonary involvement without lung involvement make up about 8% of patients with the disease [6]. There is a wide range of extrapulmonary manifestations and severity levels depending on the age, gender, and ethnicity of the patient, with African American females exhibiting the highest number of manifestations, and individuals of African descent experiencing greater organ system involvement [7].

The prevalence of renal involvement remains unclear. In autopsy studies, a granulomatous infiltrate is found in the kidneys in up to 23% and even up to 48% in small series of biopsy ﬁndings [8, 9]. The primary cause of renal dysfunction in sarcoidosis is often related to disordered calcium metabolism. Hypercalcemia, nephrocalcinosis, and nephrolithiasis are commonly observed in these patients, with nephrocalcinosis being the leading cause of progressive renal impairment. Additionally, interstitial granulomatous nephritis is the most frequently encountered histological finding in renal involvement [8]. Interstitial granulomatous nephritis is the most typical histological finding, but the development of renal insufficiency is unusual in sarcoidosis. There is a wide range of glomerulopathies associated with sarcoidosis. Different types of renal sarcoidosis have also been reported to coexist [10].

Even though sarcoidosis is a well-known disease, its renal manifestations remain elusive, and evidence regarding diagnosis and treatment is scarce. Despite difﬁculties due to limited patient numbers, future randomized controlled trials are warranted [11]. In this article, we present a case of renal manifestations of sarcoidosis, its clinical manifestations, diagnosis, and treatment options.

## Case presentation

A 12-year-old male, with no notable social or family history, presented with abdominal pain that had progressively worsened over the past month. He reported nausea, vomiting, and an unintentional weight loss of 13 kilograms over the previous five weeks. Additionally, he experienced fatigue and flank pain radiating below the umbilicus, with no other constitutional symptoms observed.

On examination, he looked ill but otherwise had normal and stable vitals. Physical examination revealed silver gray hair color (poliosis), tenderness in the right upper quadrant, and palpable liver 2 cm below the costal margin. Other examination findings were unremarkable. Laboratory investigation is shown in Table 1.

**Table 1:** Laboratory investigation upon presentation

Test	Result	Normal range
Lipase	279 U/L	12–53
Amylase	310 U/L	30–118
Calcium	2.17 mmol/l	2.08 – 2.65
Creatinine	557 µmol/l	53 – 97.2
Urea	11.2 mmol/l	3.2 – 8.2
Hemoglobin	8.4 g/dL	11.2 - 14.5
White cell count	6.26 k/ul	4.5 – 13.5
Automated lymphocytes	1.48 x10^9^/L	4 - 10.5
C-reactive protein (CRP)	12.9 mg/l	<5
C3	1.81 g/L	0.75 - 1.7
C4	0.39 g/L	0.16 - 0.48
Angiotensin-converting enzyme (ACE)	44 U/L	29 – 112
Soluble interleukin-2 receptor (sIL-2R)	19.9 ng/ml	1.9 -13.1
Antinuclear antibody (ANA)	1:80	< 1:80
Double-stranded DNA	41.5 IU/mL	<100
Microalbuminuria/creatinine ratio	102.09 mg	< 30

An ultrasound of the abdomen and pelvis revealed a well-distended gallbladder with no gallstone or wall thickening. The common bile duct and portal vein were unremarkable. The kidneys were normal in size, echogenicity, and vascularity. No stones or masses. There was no fluid, and the urinary bladder was distended. Further workups for suspicion for an autoimmune disease were unremarkable.

**Figure 1:**
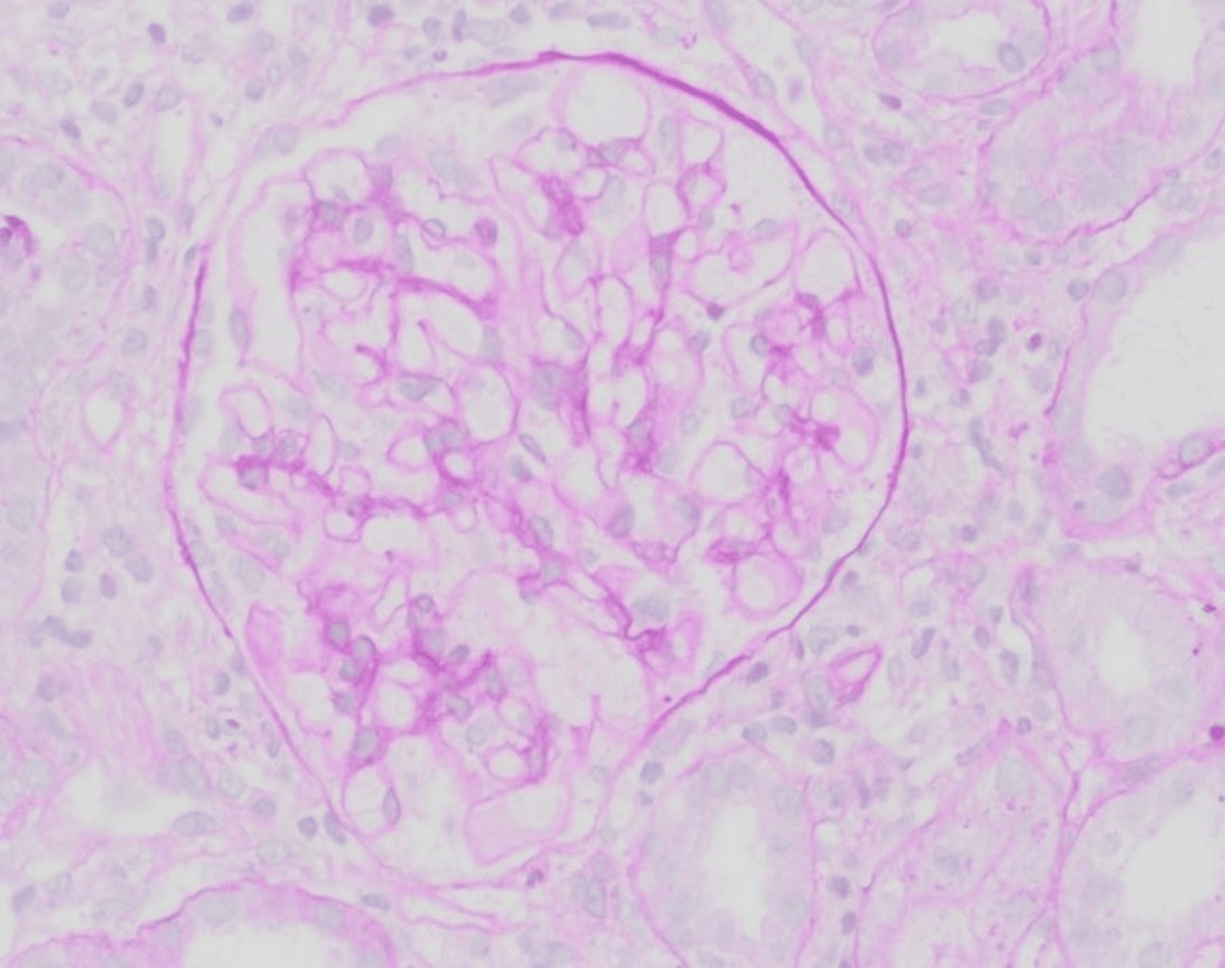
Unremarkable glomerulus

**Figure 2:**
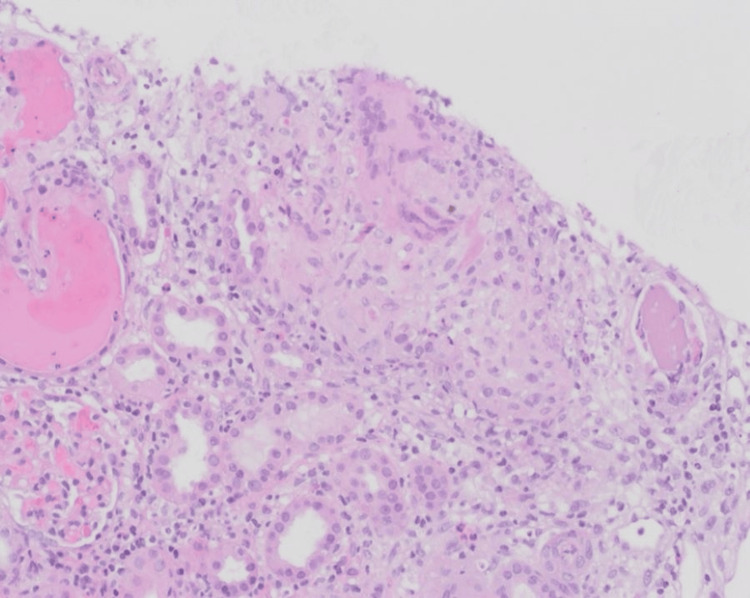
Non-necrotizing granuloma

Upon renal biopsy microscopic examination, the biopsy was examined using light microscopy and fluorescence microscopy in addition to ultrastructural evaluation by electron microscopy. A light microscopic examination was performed using the routine H&E and special stains, including periodic acid-Schiff (PAS), trichrome, and Jones silver stains. The biopsy showed cortex and medulla. Eighteen glomeruli were present. They showed unremarkable glomerular architecture with a patent capillary lumen and capillary walls of average thickness and regular structure. The interstitium showed scattered non-necrotizing granulomas with mixed mononuclear inflammatory cell infiltration but no interstitial fibrosis or tubular atrophy (Figures 1-2). The arteries and arterioles were essentially normal. The glomeruli showed negative direct immunofluorescence stains for antisera specific for IgG, IgA, IgM, C3, C1q, and kappa and lambda light chains. Glomerular ultrastructural examination by electron microscopy showed the intact podocytes' foot process, average glomerular basement membrane thickness, and architecture. There were no electron-dense deposits. The diagnosis of acute interstitial nephritis with non-necrotizing granuloma was rendered.

The genetic test showed compound heterozygous variants in the angiotensin-converting enzyme (ACE) gene, with one known pathogenic variant, ACE c.38_49del p., and the other as a variant of uncertain significance, ACE c.2110G>A p., making the diagnosis of autosomal recessive renal tubular dysgenesis less likely.

**Figure 3:**
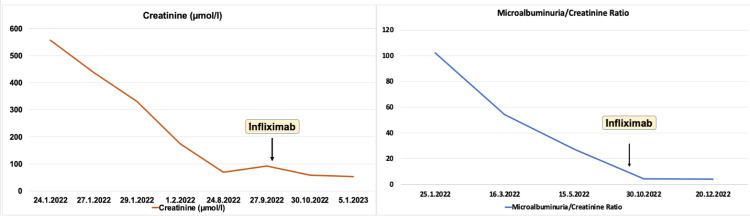
Creatinine level and microalbumin/creatinine ratio trend over the course of presentation and treatment

The patient was admitted to the pediatric nephrology unit from the emergency department. Aggressive intravenous hydration was started for pancreatitis and acute kidney injury. He received methylprednisolone pulse therapy over three days and then started on oral prednisolone as part of an acute kidney injury as well as bicarbonate sodium. Furthermore, azathioprine was added to the therapy for acute kidney injury. During follow-up, the patient developed red eyes nine months after presentation. At that time, an ophthalmology assessment was done and showed anterior granulomatous uveitis. Accordingly, the diagnosis of sarcoidosis was made. Treatment with a tumor necrosis factor-alpha (TNF-α) inhibitor infliximab was added. Creatine and microalbuminuria/creatinine ratio responded to the treatment very well (Figure 3).

## Discussion

According to our knowledge, this is the first case of juvenile sarcoidosis involving the pancreas as well as the kidney. A study identified the association between sarcoidosis and renal presentation in 39 adult patients. Of these 39 patients, 17 had confirmatory biopsy of tubule-interstitial nephritis. Changes in calcium hemostasis play a role in renal manifestations of sarcoidosis in the form of hypercalciuria. However, hypercalcemia was present in 10% of the patients [9]. A literature review published in 2021 collected 36 sarcoidosis in pediatric patients with renal involvement. The data indicate that the average age of presentation is 10.5 ± 4.6 years, with a higher prevalence in males at a ratio of 18:11. This correlates with our male patient aged 12 years. The reviewer found that renal involvement of sarcoidosis involved acute kidney injury as a common feature (86%) [12]. Furthermore, 29 of 36 patients had a mean glomerular filtration rate (GFR) of 52 ± 36 ml/min/1.73 m². Our patient, whom we discussed here, had an initially high creatinine level with a GFR of 10 mL/min/1.73 m^2^ according to the Schwartz formula. The spectrum of renal involvement includes proteinuria, hematuria, hypercalcemia, leukocyturia, and dysfunctional renal concentrating capacity [12]. Our patient did not exhibit any of these symptoms, except for proteinuria, and normal serum calcium levels were maintained throughout the course of the disease and follow-up. Bergner et al. recommended renal biopsy for patients presenting with acute kidney injury along with leukocyturia, proteinuria, or hematuria. [12]. To institute the diagnosis, a biopsy would show non-necrotizing granuloma [12]. Correspondingly, our case’s renal biopsy showed acute interstitial nephritis with non-necrotizing granuloma. In the review, two of the 36 patients had nephrocalcinosis and one had membranous nephropathy [12].

Initiation of prednisolone once tubulointerstitial nephritis is confirmed is highly recommended [11]. Methylprednisolone pulse therapy was started, and then oral prednisolone was continued; in addition, azathioprine was added, and later infliximab [13]. The patient’s creatinine level responded and normalized with treatment as shown in Figure 2. Corticosteroid therapy has magnificent effects even with patients who have advanced nephritis changes in biopsy [9]. Pancreatic involvement in sarcoidosis is described as rare [14, 15]. Nine percent of patients had a mildly elevated pancreatic amylase level [14]. The disease is usually manifested by pancreatic glands being infiltrated or compressed pancreas by the enlarged nodes around the pancreas [14]. Hypercalcemia associated with sarcoidosis plays a role in developing pancreatitis [14]. Elevated pancreatic amylase and lipase were evident in our patient. There is not enough data obtained summarize the association of pancreatitis with sarcoidosis. The relationship of chronic pancreatitis with sarcoidosis is explained as vague [16]. Disordered metabolism of calcium in the presence of sarcoidosis is witnessed [4].

Ophthalmology assessment is essential in patients with sarcoidosis. Anterior granulomatous uveitis is a common pathology involving the anterior segment of the eye [16]. Our patient's slit lamp examination by ophthalmology supported the finding of anterior uveitis.

Although the ACE enzymes were normal, we believe the variant in the ACE gene could also play a role in the diagnosis of sarcoidosis in our patient. The compound heterozygous variant in the ACE gene, as well as the clinical features, make this case worth reporting.

## Conclusions

In conclusion, to our knowledge, this case represents the first documented instance of juvenile sarcoidosis involving both the pancreas and the kidneys, highlighting the complexity and rarity of the disease in pediatric patients. The renal involvement in our patient, characterized by acute interstitial nephritis with non-necrotizing granulomas, aligns with previously reported cases, though the absence of typical symptoms such as hypercalcemia and proteinuria makes this case particularly unique. The successful response to corticosteroid therapy, supported by the use of azathioprine and infliximab, underscores the importance of early and aggressive treatment in managing severe renal manifestations of sarcoidosis. Additionally, we propose that the variant in the ACE gene may contribute to the pathogenesis and diagnosis of sarcoidosis. However, this needs further scientific research and explanation in vitro.
